# Multislice CT angiography in abdominal aortic aneurysm treated with endovascular stent grafts: evaluation of 2D and 3D visualisations

**DOI:** 10.2349/biij.3.4.e20

**Published:** 2007-10-01

**Authors:** Z Sun

**Affiliations:** Department of Imaging and Applied Physics, Curtin University of Technology, Perth, Australia

**Keywords:** Stent-graft, computed tomography, 3D reconstruction, visualisation, suprarenal fixation

## Abstract

Endovascular repair of abdominal aortic aneurysms has been introduced into the clinical practice for more than a decade and has been confirmed to be an effective alternative to conventional open surgery, especially in patients with co-morbid medical conditions. Helical CT angiography is the preferred imaging method in the follow-up of endovascular repair. Recent introduction of multislice CT scanners has augmented its diagnostic role in this area. Diagnostic value of multlislice CT has been complemented by a series of 3D post-processings, which assist vascular surgeons in accurately assessing the effect of endovascular repair by providing additional information when compared to conventional 2D axial images. These reconstructions include multiplanar reformation, curved multiplanar reconstruction, shaded surface display, maximum intensity projection, volume rendering and virtual endoscopy. This article aims to demonstrate the generation of these 2D/3D reconstructions based on multislice CT data acquired from a group of patients with abdominal aortic aneurysm following endovascular repair. A brief introduction of generating each reconstruction was provided; potential clinical applications of each reconstruction were briefly discussed. Images were presented in a dynamic format with the aim of allowing the reader to easily understand the post-processing of these reconstructions.

## INTRODUCTION

Conventional surgical repair is considered the gold standard technique in the treatment of patients with abdominal aortic aneurysm (AAA). However, it is not only an invasive technique but also carries a potential danger of perioperative and postoperative complications. Thus, less invasive methods have been investigated and endovascular repair has been confirmed to be an effective alternative since its first introduction into clinical practice in 1991 by Parodi *et al *[1-3]. Unlike conventional surgery, the success of endovascular repair mainly depends on medical imaging and multislice CT angiography (MSCTA) is considered to be the preferred modality in both pre-operative planning and post-operative follow-up [4-6]. Besides conventional 2D axial images, MSCTA has been complemented by a series of 3D reconstructions, which allows the viewer to evaluate the relationship between stent grafts and arterial branches. Moreover, dynamic visualisation has become possible with the development of CT scanning techniques and computer software. This article presents a series of reconstructed visualisations based on CTA data sets in a group of patients with AAA treated with endovascular stent grafts. These include multiplanar reformation, curved multiplanar reconstruction, shaded surface display, maximum-intensity projection, volume rendering, and virtual endoscopy. Most of the images were presented in a dynamic format with the aim of assisting readers to be familiar with variable reconstruction visualisations and help them to choose the appropriate visualisation technique when dealing with similar situations in clinical practice.

## PATIENTS’ DATA SELECTION

18 patients (15 men and 3 women, mean age: 75 years, age range: 63-84) undergoing endovascular repair of AAA were included in the study. Vascular surgeons recommended that the patients receive endovascular stent graft repair because they were unsuitable for open surgical repair due to comorbid medical conditions. All patients were treated with the Zenith AAA Endovascular Graft (William Cook Europe, Bjaeverskov, Denmark) with a suprarenal uncovered component placed above the renal arteries for obtaining proximal fixation. MSCTA was performed on a 16-slice scanner (Toshiba Medical Systems Europe, Netherlands) with the scanning protocol as follows: 1 mm section thickness, pitch 2.0 and reconstruction interval of 1 mm. MSCTA was performed with uniphasic arterial phase scans: an intravenous injection of 100 ml of non-ionic contrast media (Niopam 300, Bracco UK Ltd. High Wycombe) was administered at a rate of 2 ml/second with a scan delay of 30 seconds.

## RESULTS OF PATIENT FOLLOW-UP

The patients were routinely followed-up within one week of the stent graft implantation, and 1 month, 3 months, 6 months, 12 months and yearly thereafter. The follow-up time ranged from 24 months to 54 months, with a mean period of 40 + 7.6 months. 2D axial CT images were most commonly used to follow-up these cases and 3D reconstructions were generated to provide additional information, if required, for further assessment of potential complications. Endoleaks were found in five cases, with type I and III in one patient each, respectively, and type II in three cases. The renal function was evaluated by measuring the serum creatinine levels - it was not significantly affected in all patients, except in one patient who developed chronic renal failure because of an atrophic left renal artery and had to receive renal dialysis. Distal stent graft migration occurred in four patients and 3D image visualisations demonstrated superiority in the detection of migration, which was discussed in the following section of 3D reconstructions. All of the stent-covered aortic branches remained patent and no stenosis or occlusion was observed.

## GENERATION OF RECONSTRUCTED IMAGES

CT volume data were converted from original DICOM (Digital Imaging and Communication in Medicine) images with a commercially available software Analyze V 6.0 (www.analyzedirect.com Mayo Clinic, USA). Spatial resolution of the axial CT images was 512x512 matrix with a voxel size of 0.68 to 0.74. The data were interpolated for the purpose of generating a cubic/isotropic data set. The total number of slices within the volume of data ranged from 300 to 550 depending on the scanning coverage in each patient. As a result, a typical processed dataset had a size ranging from 40 to 65 Mbytes. Although all of the image reconstructions were performed with Analyze V 6.0 software in this study, there are a number of other commercial software packages, which allow for performance of similar post-processings.

## POST-PROCESSING OF 2D AND 3D VISUALISATIONS

### Multiplanar reformation

Multiplanar reformation (MPR) allows the user to quickly and easily view image data either in the plane of acquisition or any orthogonal plane. Movies 1 and 2 show that images were demonstrated in a sequence of coronal and sagittal MPR views in a patient with AAA after endovascular aortic repair. Besides orthogonal orientations, the intersecting sections allow the interactive display of intersecting orthogonal sections. Movie 3 shows a series of images visualised by selecting interactive sections in the same patient as in Movie 1. Planes can be interactively sliced away in the three orthogonal orientations to reveal interior sections of the cube (volume data). This enhances viewers’ understanding of the complex anatomy of aortic aneurysm and its relationship to the stent grafts.

**Movie 1:** A short video generated with MPR coronal views.

**Movie 2:** A short video generated with MPR sagittal views.

**Movie 3:** The dynamic view of orthogonal intersections.

### Curved multiplanar reconstruction

As most of the aortic aneurysms are tortuous and angulated to some extent, it is not aligned with the orthogonal axes of the 3D volume. Conventional multiplanar reformation is not always able to reveal the anatomical information required for assessment. Curved multiplanar reformation (CVR) interactively generates any arbitrary plane through a volume and allows reformatting of a series or the entire volume. This is especially useful in the assessment of patients with tortuous aneurysms as CVR allows the viewer to generate images along the centreline of the abdominal aorta. Movie 4 shows a series of CVR images in a patient after endovascular repair of AAA. The stent graft and arterial branches are clearly demonstrated in these images, even if the aneurysm is angulated because a line can be placed in the centre of the abdominal aorta, which allows generation of reconstructed images along the centre of the aorta. In this case, the suprarenal stents were deployed above the renal arteries, which appeared patent. CVR is useful in the evaluation of tortuous or angulated aortic aneurysms and the relationship between suprarenal stent struts and renal arteries.

**Movie 4:** A dynamic view of CVR reformatted coronal and sagittal images.

### Shaded surface display

Shaded surface display (SSD) represents the surface of a structure within a volume dataset. It is fast to generate because it relies on simple thresholding. It has the superiority in speed and flexibility in image rendering in the visualisation of stent graft and arterial branches. Movie 5 demonstrates a series of SSD images generated with an interval of 15^o^ along the z-axis in a patient after endovascular repair of AAA. Because of the inherent limitation of this visualisation, not all of the information in the volume data is used. Moreover, it is difficult to differentiate high-density calcification from stent wires on SSD images, as observed in the images. Thus, SSD has little role to play in the assessment of endovascular repair since it provides little additional information when compared to 2D axial images [7].

**Movie 5:** A demonstration of SSD images generated at an interval of 15° along the z-axis.

### Maximum-intensity projection

**Figure 1:**
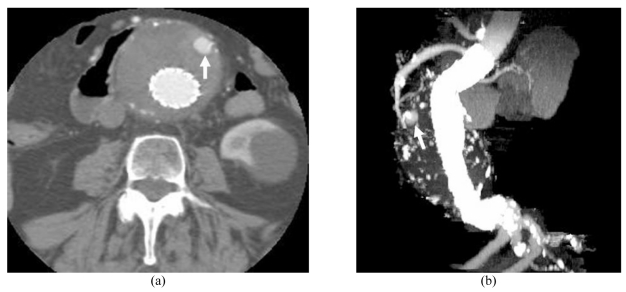
A 74-year-old man was found to develop a type II endoleak and followed-up at 24 months. The endoleak which resolved spontaneously (arrow) was clearly detected at both axial (A) and MIP images (B).

**Figure 2:**
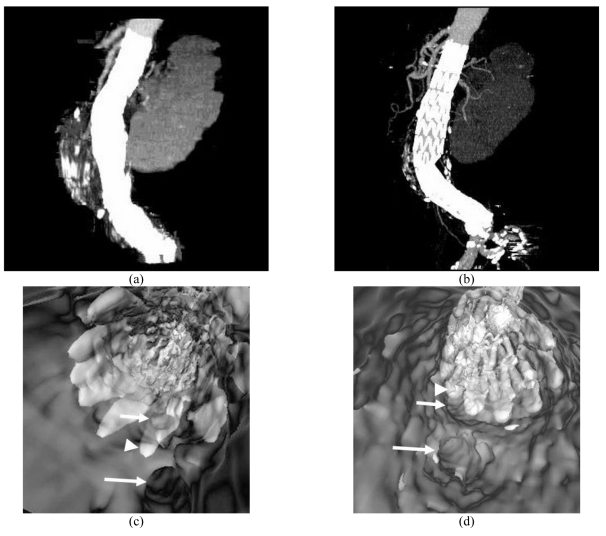
An 81-year-old woman with an abdominal aortic aneurysm was treated with a suprarenal stent graft (A) and followed-up at 36 months. A distal stent graft migration of 10.2 mm was noted in the most recent CT MIP image (B). VE (C) at 1 week of stent graft implantation showed that a stent wire crossed the SMA ostium (short arrow), but at 36 months (D), the suprarenal stent wires had shifted (short arrow). Long arrow: celiac axis, arrowhead: suprarenal stent wires.

Maximum-intensity projection (MIP) has been considered as the preferred visualisation in vascular imaging because it is independent of threshold selection and only the highest pixel value in a volume data can be displayed in the final projected images. Structures such as calcification, contrast-enhanced arteries, metal stent wires and bones are easily visualised on MIP images. Due to this reason, editing of the volume data is necessary to avoid overlapping of bony components and arteries as well as stent wires. Manual editing is time-consuming while semiautomatic/automatic editing makes post-processing a feasible and an acceptable technique in clinical practice. The Analyze software that was used in this study and other commercially available software packages for 3D post-processing allow the user to clip the volume data from any angles (x, y or z axis), which makes the generation of MIP images much quicker and more efficient. Movie 6 shows a group of MIP images generated with manual removal of the bones and soft tissue while keeping the arteries and stent wires (post-processing time is 30 minutes), whereas Movie 7 shows a similar group of MIP images generated with semi-automatic clipping method by removing some structures of the volume data from the y-axis, while the x and z-axis remain unchanged in the views (post-processing time is 3 minutes). The contrast-enhanced arteries, stent wires and kidney are clearly shown in these images, which were presented at an interval of 15^o^ along the z-axis. MIP has been found to be valuable in the follow-up of endovascular repair, e.g. detection of endoleaks and assessment of stent migration [8]. Figure 1 is an example of a patient developing endoleak after endovascular repair of AAA, while Figure 2 (A, B) is another case showing the distal migration of stent graft, which is accurately measured on sagittal MIP images.

**Movie 6:** A demonstration of MIP images generated with manual editing at an interval of 15° along the z-axis.

**Movie 7:** A demonstration of MIP images generated with clipping at an interval of 15° along the z-axis.

### Volume rendering

Different from SSD and MIP, volume rendering (VR) uses all of the information of a volume data and is able to demonstrate the anatomical structures within a volume such as calcification, aortic arterial branches and stent graft. Moreover, a colour and degree of opacity are assigned to each particular structure, which makes them easily identified and differentiated. Movie 8 shows volume rendered images displaying the variable structures within a volume with different colours in a patient following endovascular repair of AAA. As shown in the images, metal stent wires, arterial branches and bones can all be displayed in one image. VR has been accepted as the favoured 3D visualisation in assessment of the relationship between arterial branches and stent graft as it is able to demonstrate the 3D relationship between these structures and the processing time is very short, which takes only a few minutes, making it acceptable as a routine clinical tool.

**Movie 8:** A demonstration of VR images generated at an interval of 15° along the z-axis. The stent is coded with red colour while the artery branches are coded with yellow

### Virtual endoscopy

**Figure 3:**
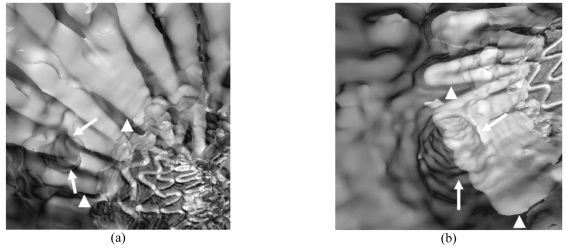
VE images show that the left renal artery (arrows in A) and SMA (arrows in B) were crossed by stent wires. Arrowheads: suprarenal stent wires.

Unlike previously mentioned 3D reconstructions, virtual endoscopy (VE) provides unique intraluminal information such as aortic ostium, intraluminal stent wires and their relation to the ostium. Moreover, based on our previous experience, the degree of encroachment to aortic ostium, mainly interference of the renal ostium by stent wires can be accurately assessed on VE images. In VE, contrast-enhanced blood needs to be removed from the vessel before performing intraluminal fly-through. The details of generation of VE images of the aortic ostia and stent wires have been described elsewhere [9]. It has been reported that VE enhances the understanding of the effect of endovascular repair on aortic branches, especially in patients with AAA treated with suprarenal stent grafts [7-10]. Figure 3 shows VE images of aortic stent wires relative to the aortic ostium. The degree and configuration of encroachment to the renal and superior mesenteric artery ostia can be clearly visualised in these images, which are valuable for vascular surgeons to assess the treatment of suprarenal stent grafting. Figure 2 (C, D) is an example showing that VE accurately detects the distal migration of stent grafts in a patient after endovascular repair of AAA. Movie 9 is a short video demonstrating the virtual fly-through in a patient with AAA pre-stent grafting, while Movie 10 shows a virtual fly-through in a post-stent grafting patient. As shown in Movie 10, the viewing point starts at the top of the suprarenal stents and moves caudally towards the bifurcated section located in the common iliac arteries.

**Movie 9:** A virtual fly-through inside the abdominal aorta in a patient pre-stent grafting. The viewing starts at the superior mesenteric artery and moves inside the aortic aneurysm.

**Movie 10:** A virtual fly-through looking at the stent wires in a patient after aortic stent grafting. The viewing starts at the top of the suprarenal stents and moves towards the bifurcated branches at common iliac arteries.

## DISCUSSION AND CONCLUSION

The role of CT angiography in aortic stent grafting has been enhanced with the introduction of MSCT. In contrast to single slice CT, CT angiography can be performed more efficiently with MSCT scanners due to faster scanning speed and higher spatial and temporal resolution [11-13]. With the latest 64-slice CT scanner, almost isotropic volume data can be obtained with MSCTA, which significantly improves the image quality of post-processing 3D reconstructions, mainly in cardiovascular imaging. Previous studies have demonstrated the diagnostic value of 3D CT visualisations in patients with AAA following endovascular repair when compared to 2D images [7-10]. However, no systematic analysis was performed with regard to the advantages and disadvantages of each image visualisation. This article has presented a series of most commonly used 2D/3D reconstruction visualisations, which were demonstrated in a dynamic format (short videos) with the aim of helping readers to be familiar with the generation and application of these visualisations in clinical practice. 3D MSCT post-processing has become a routine protocol in these authors’ daily clinical practice, and it has been widely used as an effective alternative to conventional angiography in many areas. MSCTA has replaced conventional angiography in pre-operative and post-operative assessment of endovascular repair of AAA. Therefore, accurate selection of the appropriate visualisation method is of paramount importance in clinical practice since it not only provides additional information when compared to 2D images, but also avoids unnecessary examinations and reduces the workload for imaging staff. Table 1 summarises advantages and disadvantages of these 2D and 3D visualisations in the evaluation of post-aortic stent grafting. It is noted that 2D axial images play an essential role in most of the routine follow-up, e.g. detection of endoleaks, monitoring of the aneurysm sac size, and assessment of patency of stent-covered renal arteries, which are the three main factors to determine the success of endovascular repair. However, 3D visualisations provide additional information, which is considered valuable for follow-up of endovascular repair. In comparison to 2D axial images, MIP and VE visualisations demonstrate superiority in the evaluation of stent graft migration and encroachment of aortic ostium by suprarenal stent wires. In contrast, VR is a very useful tool for demonstrating the 3D relationship between stent grafts and aortic branches. SSD has a limited role to play in post-stent grafting, while CVR is of help in patients with angulated or tortuous aneurysms. As the long-term effect of aortic stent grafting is unclear, the additional information provided by 3D visualisations in the current study is considered to be valuable for vascular surgeons to assess the outcomes of endovascular repair.

**Table 1:** Summary of the 2D and 3D visualisations in endovascular repair of AAA

**2D/3D visualisations**	**Diagnostic value of 2D/3D visualisations in common post-stent grafting complications**		
	**Aneurysm size change**	**Endoleak**	**Patency of renal arteries**
2D Axial	5	5	5
MPR	2	3	5
CVR	2	3	5
SSD	1	2	3
MIP	2	4	4
VR	1	2	5
VE	1	1	4
	**Encroachment to arteries**	**Stent graft migration**	**Renal artery stenosis / occlusion**
2D Axial	2	3	5
MPR	3	2	4
CVR	3	2	4
SSD	3	2	3
MIP	4	5	5
VR	4	3	4
VE	5	5	4

* Scores: 1 indicates the least confident, while 5 is the most confident.

In conclusion, this article has presented a series of 2D and 3D reconstruction visualisations generated from MSCT angiography based on a group of patients undergoing endovascular repair of AAA. While 2D axial CT images remain the main visualisation in most of the post-stent grafting situations, some kinds of 3D reconstructions are required to be generated for providing necessary information for accurately assessing the treatment of endovascular repair. Readers are expected to be familiar with these reconstructions and able to choose the appropriate one for diagnostic purposes while dealing with similar situations in their daily practice, especially for those heavily involved in MSCT imaging and aortic stent grafting.

## ACKNOWLEDGEMENT

The author would like to thank Dr. Peter Ellis and Dr. Anton Collins for clinical cooperation.
